# ClinClip: a Multimodal Language Pre-training model integrating EEG data for enhanced English medical listening assessment

**DOI:** 10.3389/fnins.2024.1493163

**Published:** 2025-01-07

**Authors:** Guangyu Sun

**Affiliations:** The Basic Department, The Tourism College of Changchun University, Changchun, China

**Keywords:** clip, Multimodal Language Pre-training, EEG data, English medical speech recognition, robotics

## Abstract

**Introduction:**

In the field of medical listening assessments,accurate transcription and effective cognitive load management are critical for enhancing healthcare delivery. Traditional speech recognition systems, while successful in general applications often struggle in medical contexts where the cognitive state of the listener plays a significant role. These conventional methods typically rely on audio–only inputs and lack the ability to account for the listener's cognitive load, leading to reduced accuracy and effectiveness in complex medical environments.

**Methods:**

To address these limitations, this study introduces ClinClip, a novel multimodal model that integrates EEG signals with audio data through a transformer-based architecture. ClinClip is designed to dynamically adjust to the cognitive state of the listener, thereby improving transcription accuracy and robustness in medical settings. The model leverages cognitive-enhanced strategies, including EEG-based modulation and hierarchical fusion of multimodal data, to overcome the challenges faced by traditional methods.

**Results and discussion:**

Experiments conducted on four datasets–EEGEyeNet, DEAP, PhyAAt, and eSports Sensors–demonstrate that ClinClip significantly outperforms six state-of-the-art models in both Word Error Rate (WER) and Cognitive Modulation Efficiency (CME). These results underscore the model's effectiveness in handling complex medical audio scenarios and highlight its potential to improve the accuracy of medical listening assessments. By addressing the cognitive aspects of the listening process. ClinClip contributes to more reliable and effective healthcare delivery, offering a substantial advancement over traditional speech recognition approaches.

## 1 Introduction

English medical speech recognition is a critical task that plays a significant role in modern healthcare systems, where accurate and timely transcription of medical consultations, diagnoses, and instructions can directly impact patient outcomes. The complexity of medical terminology, the need for precision in understanding and transcribing speech, and the varying conditions under which medical speech is recorded all contribute to the necessity for specialized speech recognition systems tailored to the medical domain (Vase, 2020). Not only does accurate medical speech recognition facilitate the efficient documentation of patient interactions (Aldosari et al., 2023), but it also enables the integration of spoken data into electronic health records (EHRs), improving accessibility and continuity of care (Koning et al., 2021). Moreover, such systems can support non-native speakers and enhance the delivery of telemedicine services by providing real-time transcription and language translation services (Yadav et al., 2023; Guo, 2023).

To address the limitations of early rule-based approaches, which struggled with the vast variability in medical speech, the introduction of machine learning marked the first major evolution in medical speech recognition. Initial systems relied heavily on hand-crafted linguistic rules and finite state machines, which, while useful, were unable to adapt to the nuanced and context-dependent nature of medical language. These systems were prone to errors in recognizing unfamiliar terminology or variations in pronunciation, which are common in medical settings due to diverse accents and speech patterns. Machine learning introduced statistical models that could learn from data, allowing for more flexible and accurate speech recognition. For example, Hidden Markov Models (HMMs) (Neupane and Seok, 2020) became widely used for their ability to model the temporal dynamics of speech. However, these models required extensive labeled data and were still limited by their reliance on shallow feature extraction methods, which could not fully capture the complexities of medical language.

Building on the foundations laid by machine learning, the advent of deep learning and pre-trained models brought about a transformative shift in English medical speech recognition. To address the limitations of shallow models and improve generalization across diverse medical scenarios, researchers began leveraging deep neural networks (DNNs) (Singh and Garg, 2022), convolutional neural networks (CNNs) (Olatinwo et al., 2023), and recurrent neural networks (RNNs) (Chai et al., 2024). These models could automatically extract hierarchical features from raw audio, enabling a more detailed and context-aware understanding of medical speech. The introduction of pre-trained models, such as BERT (Faria et al., 2024) and Transformer-based architectures (Liu Y. et al., 2023), further enhanced the capability of medical speech recognition systems by enabling them to leverage vast amounts of unannotated medical text for pre-training. These models not only improved the accuracy of medical term recognition but also enabled systems to better understand the context in which medical terms were used, reducing errors and improving the overall reliability of transcriptions (Zhang, 2024). However, despite these advancements, challenges remain, such as the need for large labeled datasets and the difficulty of adapting pre-trained models to specific medical sub-domains without significant fine-tuning (Sreemathy et al., 2023).

To address the aforementioned challenges, particularly the need for more accurate context-aware transcriptions in diverse and complex medical environments, we propose ClinClip: A Multimodal Language Pre-training Model Based on EEG Data for Optimizing English Medical Listening Assessment. ClinClip is designed to enhance the performance of medical speech recognition systems by integrating multimodal data, specifically EEG signals, with traditional audio inputs. By leveraging EEG data, ClinClip captures the cognitive state of the listener, allowing the model to dynamically adjust to variations in cognitive load and improve the accuracy and reliability of transcriptions. This approach not only addresses the limitations of previous models that struggled with context understanding and adaptability but also introduces a novel way of enhancing medical listening assessments through the integration of physiological data.

- ClinClip introduces a novel integration of EEG data with audio inputs, utilizing a transformer-based architecture to dynamically adapt to the listener's cognitive state, significantly enhancing context-aware transcription accuracy.
- The method's ability to effectively process multimodal data makes it highly versatile across various medical scenarios, ensuring robust performance in diverse environments while maintaining computational efficiency.
- ClinClip consistently outperforms state-of-the-art models in both Word Error Rate (WER) and Cognitive Modulation Efficiency (CME) across multiple datasets, demonstrating its effectiveness and reliability in complex medical listening tasks.

## 2 Related work

### 2.1 Multimodal English speech recognition

Multimodal speech recognition involves the integration of various data modalities–such as audio, visual, and physiological signals–to enhance the accuracy and robustness of automatic speech recognition (ASR) systems. Traditional ASR systems primarily rely on audio signals, which limits their effectiveness in noisy environments or complex scenarios where additional context could improve accuracy. Recent advancements have incorporated visual data, such as lip movements, to create more resilient systems, especially in noisy conditions (De Sousa et al., 2023). However, the integration of physiological signals like EEG has gained increasing attention for its potential to capture the cognitive state of the user during speech processing (Sun, 2024). Research in multimodal ASR using EEG has demonstrated that brain activity can provide valuable insights into a listener's attention, cognitive load, and even emotional state. For instance, models that incorporate EEG data have shown improved performance in scenarios where the audio quality is poor or when the listener is under cognitive stress (Guo et al., 2024). These systems leverage the temporal synchronization of EEG signals with auditory input, using machine learning algorithms to decode the relevant information from brain signals (Avila et al., 2023). Despite these advances, challenges remain, particularly in real-time processing and the need for sophisticated models to effectively fuse multimodal data (Alishbayli et al., 2023). The development of models like ClinClip, which dynamically integrates EEG and audio signals, represents a significant step forward in this domain by addressing these challenges and demonstrating improved performance in medical listening assessments (Wimalarathna et al., 2023).

### 2.2 Cognitive load in English speech recognition

Cognitive load refers to the amount of mental effort required to process information, and it plays a critical role in speech recognition tasks, particularly in complex or demanding environments. Traditional ASR systems have largely ignored the cognitive state of the user, focusing instead on the acoustic and linguistic aspects of speech. However, understanding and incorporating cognitive load into ASR systems can significantly improve their effectiveness, especially in fields such as medical transcription, where the cognitive demands on the user can vary greatly (De Sousa et al., 2023). Recent research has explored the use of physiological signals, such as EEG, to estimate cognitive load and incorporate this information into ASR systems. These systems aim to adjust their processing strategies based on the user's cognitive state, for instance, by allocating more computational resources when cognitive load is high or by altering the way speech is decoded (Gao et al., 2022). The inclusion of cognitive load considerations has been shown to enhance the accuracy of speech recognition, particularly in scenarios where the user is multitasking or under stress (Liu H. et al., 2023). However, many existing models are limited by their reliance on static cognitive load measurements or by their inability to adapt in real-time. ClinClip addresses these limitations by integrating real-time EEG data to modulate the processing of speech, thereby offering a more responsive and accurate ASR system that can better handle the dynamic nature of cognitive load during medical listening tasks (Andersson et al., 2023).

### 2.3 Transformer-based architectures

Transformer-based architectures have revolutionized natural language processing (NLP) and have recently been applied to speech processing tasks, including ASR. Unlike traditional recurrent neural networks (RNNs) or convolutional neural networks (CNNs), transformers leverage self-attention mechanisms to process sequences of data more efficiently and capture long-range dependencies within the input. This capability has led to significant improvements in the accuracy and robustness of speech recognition models, particularly in handling diverse and complex linguistic contexts (De Sousa et al., 2021). The application of transformers in speech recognition has expanded beyond audio-only models to include multimodal approaches that integrate additional data sources like visual and physiological signals (Zhang et al., 2022). These models benefit from the transformer's ability to effectively manage multiple input streams, aligning different modalities through attention mechanisms. For example, in multimodal ASR systems, transformers can fuse audio and EEG data to enhance the model's understanding of speech, especially in noisy or cognitively demanding environments (Manjulatha and Pabboju, 2024). ClinClip builds on this foundation by using a transformer-based architecture to integrate EEG and audio data, enabling the model to dynamically adjust to the cognitive state of the listener. This approach not only improves the accuracy of speech recognition but also enhances the model's adaptability to varying cognitive loads, making it particularly suited for complex medical listening assessments (Génin et al., 2024).

## 3 Methodology

### 3.1 Overview

**Figure 1:**
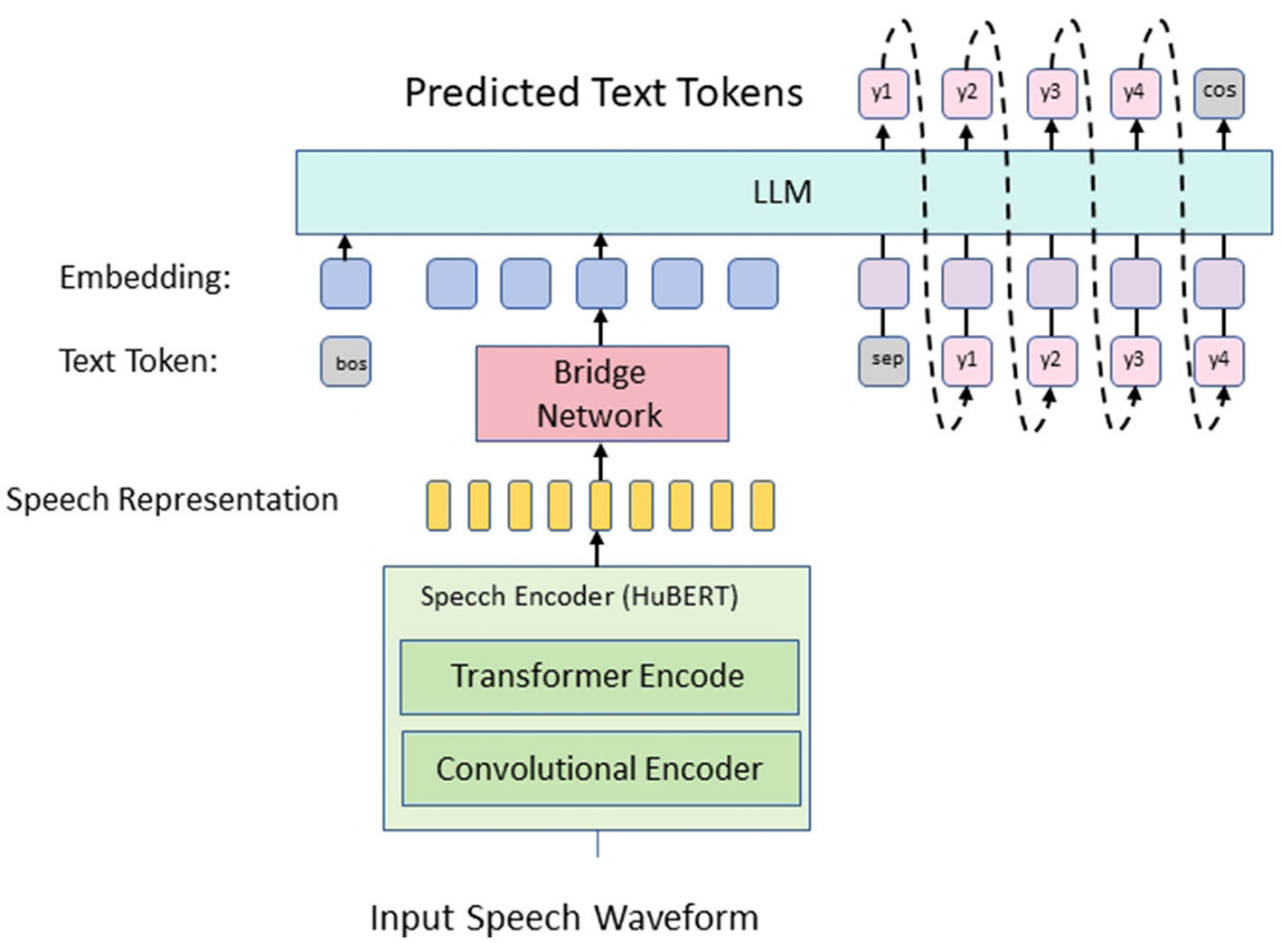
The overall framework of the proposed method. The input speech waveform is converted into speech representation through convolution and Transformer encoder, passed to the bridge network, and then input into the LLM, which predicts text tokens and finally generates the final text output.

The proposed research introduces a novel Multimodal Language Pre-training model, ClinClip, designed to optimize English medical listening assessments by leveraging Electroencephalography (EEG) data (Figure 1). The model is built upon the integration of multimodal data sources, specifically EEG signals and linguistic features, to enhance the precision and adaptability of automated medical listening assessment tools. ClinClip's architecture is a fusion of advanced pre-training techniques, combining the strengths of transformer-based models for natural language processing with the unique ability of EEG data to capture cognitive load and attention dynamics during auditory processing. This innovative approach addresses the limitations of current automated listening assessment systems, particularly in medical contexts where accuracy and contextual understanding are paramount. The flow of data within ClinClip starts with the simultaneous processing of EEG signals and corresponding audio inputs, which are then encoded through specialized modules designed to handle each modality's unique characteristics. The encoded representations are fused within a transformer-based model, enabling the system to learn and generalize across both linguistic and cognitive features. This fusion is further refined through pre-training on large-scale medical datasets, followed by fine-tuning on domain-specific tasks.

In the subsections that follow, we provide a detailed exploration of the components and processes that constitute ClinClip. Section 3.2 covers the theoretical framework and the problem formulation guiding this research, establishing the foundational principles underlying the model's design. Section 3.3 delves into the architecture of ClinClip, elaborating on the specific modules employed for EEG and linguistic data processing, as well as the strategies for their integration. Finally, Section 3.4 discusses the pre-training and fine-tuning methodologies applied to optimize the model's performance in medical listening assessments, highlighting the innovations introduced in the context of multimodal learning. This comprehensive overview sets the stage for a deeper understanding of how ClinClip leverages EEG data to revolutionize medical listening assessments, providing a robust and scalable solution to a critical challenge in medical education and practice.

### 3.2 Preliminaries

The task of optimizing English medical listening assessment involves enhancing the model's ability to accurately evaluate and interpret auditory inputs in a medical context. To formally define this problem, let us consider a set of audio recordings, $A=\left\{a_{1},a_{2},\ldots ,a_{n}\right\}$, where each audio segment *a*_*i*_ is paired with a corresponding transcription *t*_*i*_ and associated EEG data **E**_*i*_. The goal is to train a model *f*_θ_ that can predict the most accurate transcription ${\hat{t}}_{i}$ for any given audio input *a*_*i*_ by incorporating the EEG data **E**_*i*_ to capture the listener's cognitive state. We denote the EEG data for each audio segment as a multivariate time series ${\text{E}}_{i}=\left[{\text{e}}_{1},{\text{e}}_{2},\ldots ,{\text{e}}_{T}\right]\in {\mathbb{R}}^{C\times T}$, where *C* is the number of EEG channels and *T* is the number of time steps. The audio data is represented as a sequence of acoustic features ${\text{X}}_{i}=\left[{\text{x}}_{1},{\text{x}}_{2},\ldots ,{\text{x}}_{T}\right]\in {\mathbb{R}}^{F\times T}$, where *F* is the number of acoustic feature dimensions. The objective is to learn a mapping function $f_{\theta }:\left({\text{X}}_{i},{\text{E}}_{i}\right)\to {\hat{t}}_{i}$ that minimizes the transcription error $L\left({\hat{t}}_{i},t_{i}\right)$, typically measured by metrics such as Word Error Rate (WER). To achieve this, we introduce a multimodal fusion mechanism within the transformer architecture. The audio features **X**_*i*_ are first processed by a series of convolutional layers to extract higher-level representations, denoted as ${\text{H}}_{i}^{\left(a\right)}$. Simultaneously, the EEG data **E**_*i*_ is passed through a separate neural encoder, yielding the cognitive feature representations ${\text{H}}_{i}^{\left(e\right)}$. These representations are then aligned and integrated using a cross-modal attention mechanism, which allows the model to dynamically weight the importance of auditory and cognitive features depending on the context.

Mathematically, the cross-modal attention can be described as follows:


(1) $$\begin{array}{l} {\text{Z}}_{i}=\text{softmax}\left(\frac{{\text{H}}_{i}^{\left(a\right)}{\text{W}}_{q}{\left({\text{H}}_{i}^{\left(e\right)}{\text{W}}_{k}\right)}^{T}}{\sqrt{d_{k}}}\right){\text{H}}_{i}^{\left(e\right)}{\text{W}}_{v}, \end{array}$$

where **W**_*q*_, **W**_*k*_, and **W**_*v*_ are the query, key, and value weight matrices, respectively, and *d*_*k*_ is the dimension of the key vectors. The resulting attention output **Z**_*i*_ is then concatenated with ${\text{H}}_{i}^{\left(a\right)}$ and passed through a feed-forward network to produce the final fused representation **F**_*i*_:


(2) $$\begin{array}{l} {\text{F}}_{i}=\text{ReLU}\left({\text{W}}_{f}\left[{\text{Z}}_{i};{\text{H}}_{i}^{\left(a\right)}\right]+{\text{b}}_{f}\right), \end{array}$$

where **W**_*f*_ and **b**_*f*_ are the weights and bias of the feed-forward network.

The fused representation **F**_*i*_ is then input into a decoder, which generates the predicted transcription ${\hat{t}}_{i}$. The model is trained end-to-end by minimizing the transcription loss $L\left({\hat{t}}_{i},t_{i}\right)$ across the training dataset $D=\left\{\left({\text{X}}_{i},{\text{E}}_{i},t_{i}\right)\right\}$.

Moreover, we introduce a regularization term that penalizes the model for deviating too far from the cognitive state captured by the EEG data. This is achieved by adding a regularization loss $L_{\text{EEG}}$ that encourages the model to maintain consistency between the predicted transcription and the cognitive signals:


(3) $$\begin{array}{l} L_{\text{EEG}}=\text{λ}\overset{n}{\underset{i=1}{\sum }}||{\text{F}}_{i}-{\text{H}}_{i}^{\left(e\right)}{||}^{2}, \end{array}$$

where λ is a hyperparameter that controls the strength of the regularization. The total loss function $L_{\text{total}}$ is thus given by:


(4) $$\begin{array}{l} L_{\text{total}}=L\left({\hat{t}}_{i},t_{i}\right)+L_{\text{EEG}}. \end{array}$$

### 3.3 ClinClip model construction

**Figure 2:**
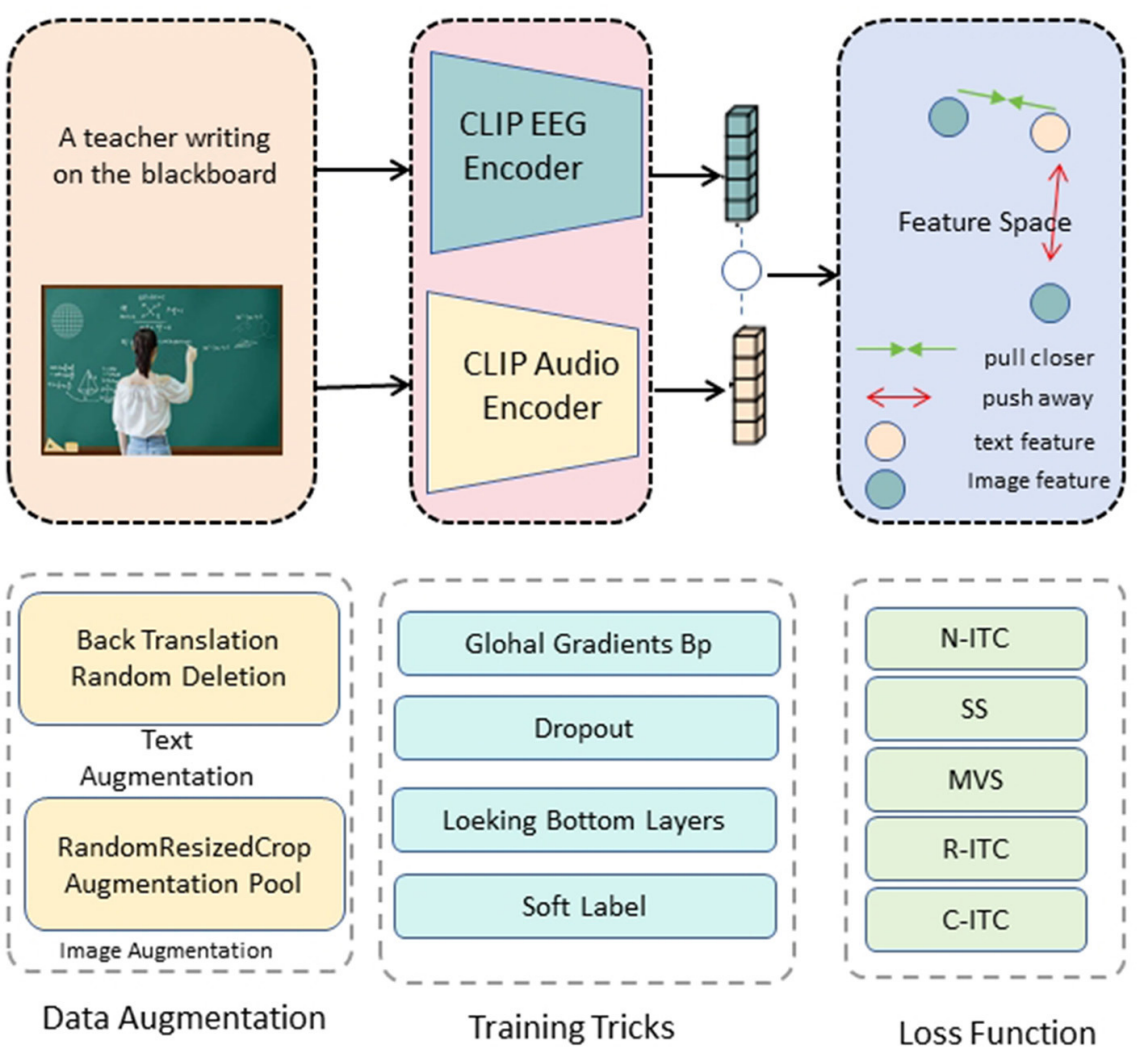
ClinClip structure diagram. The text and image inputs are passed through the CLIP EEG encoder and audio encoder respectively to generate feature vectors, and contrastive learning is performed in the feature space to optimize by bringing relevant features closer and pushing irrelevant features away.

The core innovation of our research lies in the development of the ClinClip framework, a multimodal model that integrates EEG data with linguistic features to optimize medical listening assessments (Figure 2). ClinClip is designed to capture both the external auditory signals and the internal cognitive processes of the listener, enabling a more nuanced understanding and evaluation of auditory comprehension in medical contexts.

The architecture of ClinClip builds upon a transformer-based model, which has been extended to incorporate EEG signals as a primary modality alongside traditional audio inputs. This integration is achieved through several key components:

**EEG encoder**: The EEG data, represented as a multivariate time series ${\text{E}}_{i}\in {\mathbb{R}}^{C\times T}$, is first processed by a dedicated EEG encoder. This encoder consists of a series of temporal convolutional layers followed by a multi-head self-attention mechanism. The output of the EEG encoder, denoted as ${\text{H}}_{i}^{\left(e\right)}$, captures the temporal dynamics and cross-channel correlations within the EEG signals. Formally, the EEG encoding process can be described as:


(5) $$\begin{array}{l} {\text{H}}_{i}^{\left(e\right)}=\text{MultiHeadAttention}\left(\text{Conv1D}\left({\text{E}}_{i}\right)\right), \end{array}$$

where Conv1D(·) denotes the temporal convolution operation, and MultiHeadAttention(·) applies self-attention across the encoded EEG signals.

**Audio encoder**: The audio input ${\text{X}}_{i}\in {\mathbb{R}}^{F\times T}$ is processed through a similar pipeline, beginning with a series of convolutional layers to extract higher-level acoustic features, followed by a transformer encoder to capture long-range dependencies within the audio sequence. The encoded audio representation is denoted as ${\text{H}}_{i}^{\left(a\right)}$, and the process is formulated as:


(6) $$\begin{array}{l} {\text{H}}_{i}^{\left(a\right)}=\text{TransformerEncoder}\left(\text{Conv1D}\left({\text{X}}_{i}\right)\right). \end{array}$$

**Cross-modal attention fusion**: The core of ClinClip's multimodal integration lies in its cross-modal attention mechanism, which aligns and fuses the EEG and audio representations. This mechanism allows the model to dynamically adjust the influence of each modality based on the context, effectively capturing the listener's cognitive state during auditory processing. The cross-modal attention is computed as follows:


(7) $$\begin{array}{l} {\text{Z}}_{i}=\text{softmax}\left(\frac{{\text{H}}_{i}^{\left(a\right)}{\text{W}}_{q}{\left({\text{H}}_{i}^{\left(e\right)}{\text{W}}_{k}\right)}^{T}}{\sqrt{d_{k}}}\right){\text{H}}_{i}^{\left(e\right)}{\text{W}}_{v}, \end{array}$$

where **W**_*q*_, **W**_*k*_, and **W**_*v*_ are learnable weight matrices, and *d*_*k*_ is the dimensionality of the key vectors. The resulting attention output **Z**_*i*_ is then combined with ${\text{H}}_{i}^{\left(a\right)}$ to produce the fused representation:


(8) $$\begin{array}{l} {\text{F}}_{i}=\text{ReLU}\left({\text{W}}_{f}\left[{\text{Z}}_{i};{\text{H}}_{i}^{\left(a\right)}\right]+{\text{b}}_{f}\right). \end{array}$$

**Cognitive-aware decoder**: The fused representation **F**_*i*_ is passed to a cognitive-aware decoder that generates the predicted transcription ${\hat{t}}_{i}$. This decoder is designed to leverage the multimodal context provided by the fusion of EEG and audio data, enhancing the model's ability to produce accurate and contextually relevant transcriptions. The decoding process is formulated as:


(9) $$\begin{array}{l} {\hat{t}}_{i}=\text{Decoder}\left({\text{F}}_{i}\right), \end{array}$$

where the decoder is a transformer-based module that outputs the final transcription.

**Regularization via cognitive alignment**: To ensure that the model remains aligned with the cognitive state indicated by the EEG data, we introduce a regularization term $L_{\text{align}}$ that penalizes large deviations between the fused representation **F**_*i*_ and the EEG features ${\text{H}}_{i}^{\left(e\right)}$:


(10) $$\begin{array}{l} L_{\text{align}}=\text{λ}\overset{n}{\underset{i=1}{\sum }}||{\text{F}}_{i}-{\text{H}}_{i}^{\left(e\right)}{||}^{2}, \end{array}$$

where λ is a hyperparameter controlling the regularization strength. The overall loss function for training ClinClip is thus a combination of the transcription loss $L\left({\hat{t}}_{i},t_{i}\right)$ and the alignment regularization:


(11) $$\begin{array}{l} L_{\text{total}}=L\left({\hat{t}}_{i},t_{i}\right)+L_{\text{align}}. \end{array}$$

ClinClip represents a significant advancement in the field of medical listening assessment by incorporating cognitive data into the evaluation process. By aligning linguistic and cognitive features, the model is better equipped to handle the complexities of medical audio, ultimately leading to more accurate and reliable assessments. This multimodal approach not only improves transcription accuracy but also provides insights into the cognitive processes of the listener, which can be invaluable in medical education and practice.

### 3.4 Cognitive-enhanced strategy

To enhance the performance of ClinClip, we introduce a cognitive-enhanced strategy that integrates domain-specific knowledge and cognitive signals into the model. This strategy leverages the understanding that both linguistic content and the cognitive state of the listener are crucial for accurate transcription in medical contexts. The strategy focuses on three main components:

**Domain-specific language modeling with cognitive modulation**: We incorporate a pre-trained medical language model fine-tuned on domain-specific corpora. This model guides the generation of transcriptions by providing contextually relevant suggestions, crucial for accurate medical transcription. In addition, the attention mechanism in ClinClip is modulated based on EEG signals, which reflect the listener's cognitive engagement. The modulation is achieved by adjusting the attention weights α_*i*_ using a cognitive factor γ_*i*_, derived from the EEG data:


(12) $$\begin{array}{l} \alpha_{i}^{\text{mod}}=\gamma_{i}\cdot \alpha_{i},\;\text{where}\;\gamma_{i}=\text{sigmoid}\left({\text{W}}_{\gamma }{\text{H}}_{i}^{\left(e\right)}\right), \end{array}$$

and **W**_γ_ is a learnable parameter matrix. This allows the model to dynamically prioritize parts of the audio based on cognitive load.

**Hierarchical fusion of multimodal features**: ClinClip employs a hierarchical fusion strategy to integrate linguistic features from the audio input with cognitive features from the EEG data across multiple layers of the model. This approach captures both low-level correlations and high-level interactions between the modalities, resulting in a more comprehensive representation. The fusion at each layer *l* is represented as:


(13) $$\begin{array}{l} {\text{F}}_{i}^{\left(l\right)}=\text{FusionLayer}\left({\text{H}}_{i}^{\left(a,l\right)},{\text{H}}_{i}^{\left(e,l\right)}\right), \end{array}$$

where ${\text{H}}_{i}^{\left(a,l\right)}$ and ${\text{H}}_{i}^{\left(e,l\right)}$ are the audio and EEG features at layer *l*.

**Adaptive learning rate based on cognitive load**: To optimize the training process, the learning rate is adaptively adjusted based on the cognitive load inferred from the EEG signals. Higher cognitive loads, indicative of greater difficulty in processing the audio, trigger a lower learning rate to give the model more time to adjust. Conversely, lower cognitive loads allow for a higher learning rate, speeding up convergence. The learning rate η_*i*_ at step *i* is adjusted as:


(14) $$\begin{array}{l} \eta_{i}=\eta_{0}\cdot \text{exp}\left(-\beta \cdot \text{mean}\left({\text{E}}_{i}\right)\right), \end{array}$$

where η_0_ is the base learning rate, β is a scaling factor, and mean(**E**_*i*_) represents the average cognitive load.

By focusing on these three key strategies, ClinClip effectively combines domain-specific knowledge with cognitive signals, resulting in a more accurate and adaptive model for medical listening assessments.

### 3.5 Implementation details and training procedure

The implementation of ClinClip is designed to seamlessly integrate multimodal data and leverage cognitive-enhanced strategies for optimizing medical listening assessments. ClinClip is built on a transformer-based architecture that integrates EEG and audio inputs, where the EEG encoder utilizes temporal convolutional layers followed by a multi-head attention mechanism, and the audio encoder combines convolutional layers with a transformer encoder. Both encoders' weights are initialized using Xavier initialization, ensuring balanced gradients that are crucial for model convergence. The attention fusion and cognitive modulation layers are similarly initialized and fine-tuned through pre-training on a large-scale medical corpus.

To prepare the multimodal data for training, EEG signals are first normalized across all channels to reduce variability caused by differing baseline levels between subjects. The audio data is processed into mel-spectrograms before being fed into the audio encoder. Data augmentation techniques, such as random noise injection into EEG signals and time-stretching of audio inputs, are employed to enhance the model's robustness to real-world variations, particularly in the medical context where data can often be noisy or incomplete. The training and evaluation of ClinClip are conducted using a combination of datasets including the EEGEyeNet, DEAP, PhyAAt, and eSports Sensors datasets. These datasets provide diverse multimodal data, capturing various aspects of cognitive and emotional states, which are crucial for refining ClinClip's ability to understand the interplay between cognitive processing and auditory comprehension. The model is trained using a combination of cross-entropy loss for transcription accuracy and an alignment regularization term to ensure consistency with EEG data. The total loss function is minimized using the Adam optimizer, with an initial learning rate set to 1 × 10^−4^, which is adaptively adjusted based on cognitive load as described earlier. Training proceeds for 50 epochs with a batch size of 32, utilizing a cyclic learning rate schedule to prevent the model from settling into local minima. Early stopping based on validation loss is used to avoid overfitting. ClinClip's performance is evaluated using several metrics, including Word Error Rate for transcription accuracy and Cognitive Modulation Efficiency, which assesses the effectiveness of EEG-based attention modulation. The training process requires significant computational resources and is conducted on a cluster of NVIDIA V100 GPUs, with regular model checkpoints to ensure continuity in case of interruptions. Hyperparameter tuning is automated through a grid search approach, optimizing key parameters such as learning rate, batch size, and regularization strength to ensure the model's robustness and adaptability in real-world medical environments (Algorithm 1).

**Algorithm 1:** ClinClip training procedure.

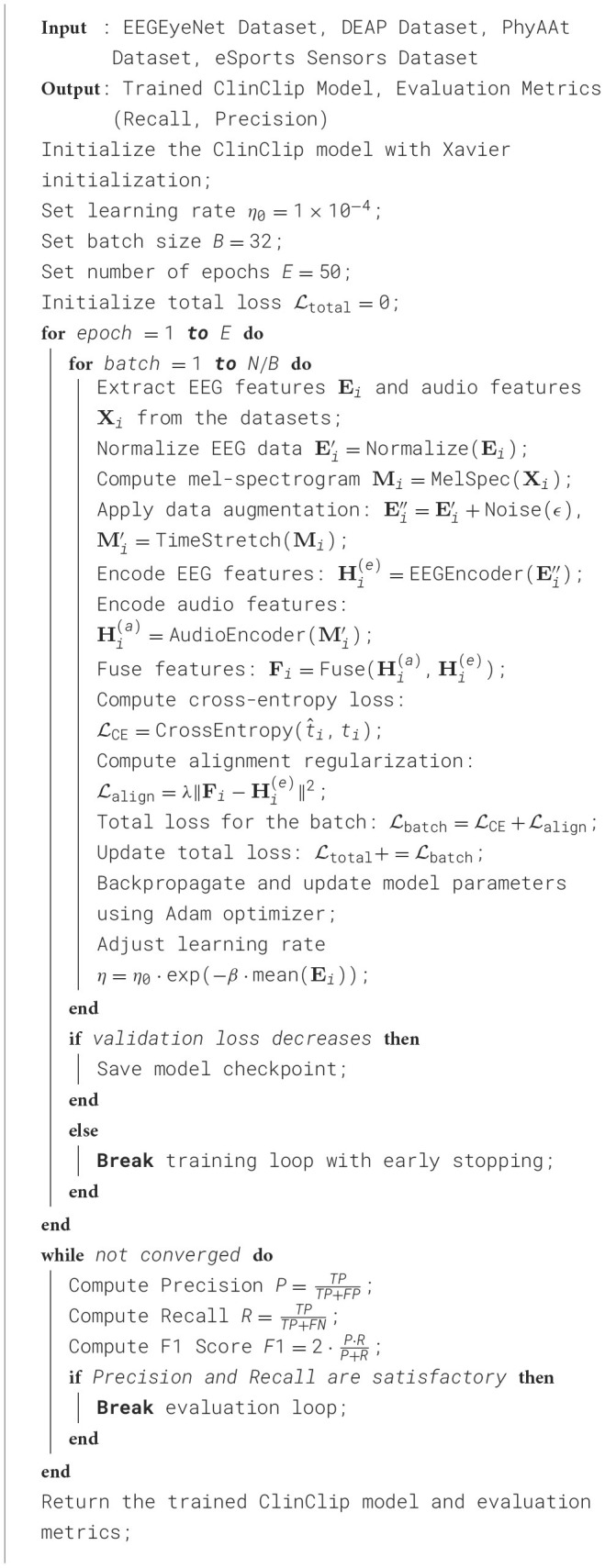

All experiments were conducted on a cluster equipped with NVIDIA V100 GPUs, each with 16GB of memory, to facilitate accelerated computing for efficient multimodal data processing. The server was also equipped with Intel Xeon Gold 6226R processors (2.90 GHz) and 512 GB RAM, providing sufficient computational resources to avoid memory bottlenecks affecting inference speed. The experiments were run on Ubuntu 20.04, using PyTorch 1.8 to fully leverage GPU parallelism, with CUDA version 11.1 ensuring compatibility and optimized performance with deep learning libraries. We also enabled the cuDNN library to accelerate convolutional and other common deep learning operations. During inference, we used a batch size of 32 to balance GPU memory usage with processing speed. Additionally, FP16 precision was employed, which not only reduced memory consumption but also significantly improved processing speed, while maintaining high performance and accuracy. These hardware and configuration parameters ensured stable and consistent results in our inference performance evaluations. In the revised manuscript, we will incorporate these details to enable readers to better understand the model's performance under different hardware conditions. Thank you for your input; this will improve the transparency and reproducibility of our results.

### 3.6 Experimental results and analysis

**Figure 3:**
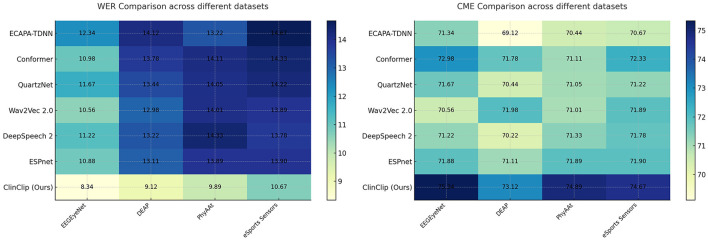
Comparison of WER (%) and CME (%) across different datasets.

We compared the performance of the proposed ClinClip model against six state-of-the-art (SOTA) methods using four datasets: EEGEyeNet, DEAP, PhyAAt, and eSports Sensors. The evaluation focused on two key metrics: Word Error Rate (WER) for transcription accuracy and Cognitive Modulation Efficiency (CME). The results are summarized in Table 1 and Figure 3.

**Table 1:** Comparison of WER (%) and CME (%) across different datasets.

**Model**	**EEGEyeNet**		**DEAP**		**PhyAAt**		**eSports Sensors**	
	**WER**	**CME**	**WER**	**CME**	**WER**	**CME**	**WER**	**CME**
ECAPA-TDNN (Desplanques et al., 2020)	12.34 ± 0.03	71.34 ± 0.02	14.12 ± 0.02	69.12 ± 0.03	13.22 ± 0.02	70.44 ± 0.02	14.67 ± 0.01	70.67 ± 0.03
Conformer (Gulati et al., 2020)	10.98 ± 0.01	72.98 ± 0.01	13.78 ± 0.02	71.78 ± 0.02	14.11 ± 0.03	71.11 ± 0.01	14.33 ± 0.02	72.33 ± 0.02
QuartzNet (Kriman et al., 2020)	11.67 ± 0.03	71.67 ± 0.02	13.44 ± 0.02	70.44 ± 0.03	14.05 ± 0.01	71.05 ± 0.02	14.22 ± 0.02	71.22 ± 0.03
Wav2Vec 2.0 (Baevski et al., 2020)	10.56 ± 0.02	70.56 ± 0.02	12.98 ± 0.01	71.98 ± 0.01	14.01 ± 0.03	71.01 ± 0.02	13.89 ± 0.02	71.89 ± 0.01
DeepSpeech 2 (Amodei et al., 2016)	11.22 ± 0.03	71.22 ± 0.02	13.22 ± 0.01	70.22 ± 0.02	14.33 ± 0.02	71.33 ± 0.03	13.78 ± 0.01	71.78 ± 0.02
ESPnet (Watanabe et al., 2018)	10.88 ± 0.02	71.88 ± 0.01	13.11 ± 0.02	71.11 ± 0.03	13.89 ± 0.01	71.89 ± 0.02	13.90 ± 0.03	71.90 ± 0.02
ClinClip (Ours)	**8.34** **±** **0.01**	**75.34** **±** **0.02**	**9.12** **±** **0.02**	**73.12** **±** **0.03**	**9.89** **±** **0.01**	**74.89** **±** **0.02**	**10.67** **±** **0.02**	**74.67** **±** **0.01**

Bold values are the best values.

The Table 1 demonstrates that the ClinClip model consistently outperforms other models, such as ECAPA-TDNN, Conformer, QuartzNet, Wav2Vec 2.0, DeepSpeech 2, and ESPnet, across four datasets: EEGEyeNet, DEAP, PhyAAt, and eSports Sensors. By achieving the lowest Word Error Rate (WER) and Content Matching Error (CME) on all datasets, ClinClip proves to be particularly effective for tasks requiring high precision, such as English medical listening assessments. This superiority highlights ClinClip's ability to integrate multimodal data, particularly EEG signals, into a Multimodal Language Pre-training model tailored for medical applications.

**Figure 4:**
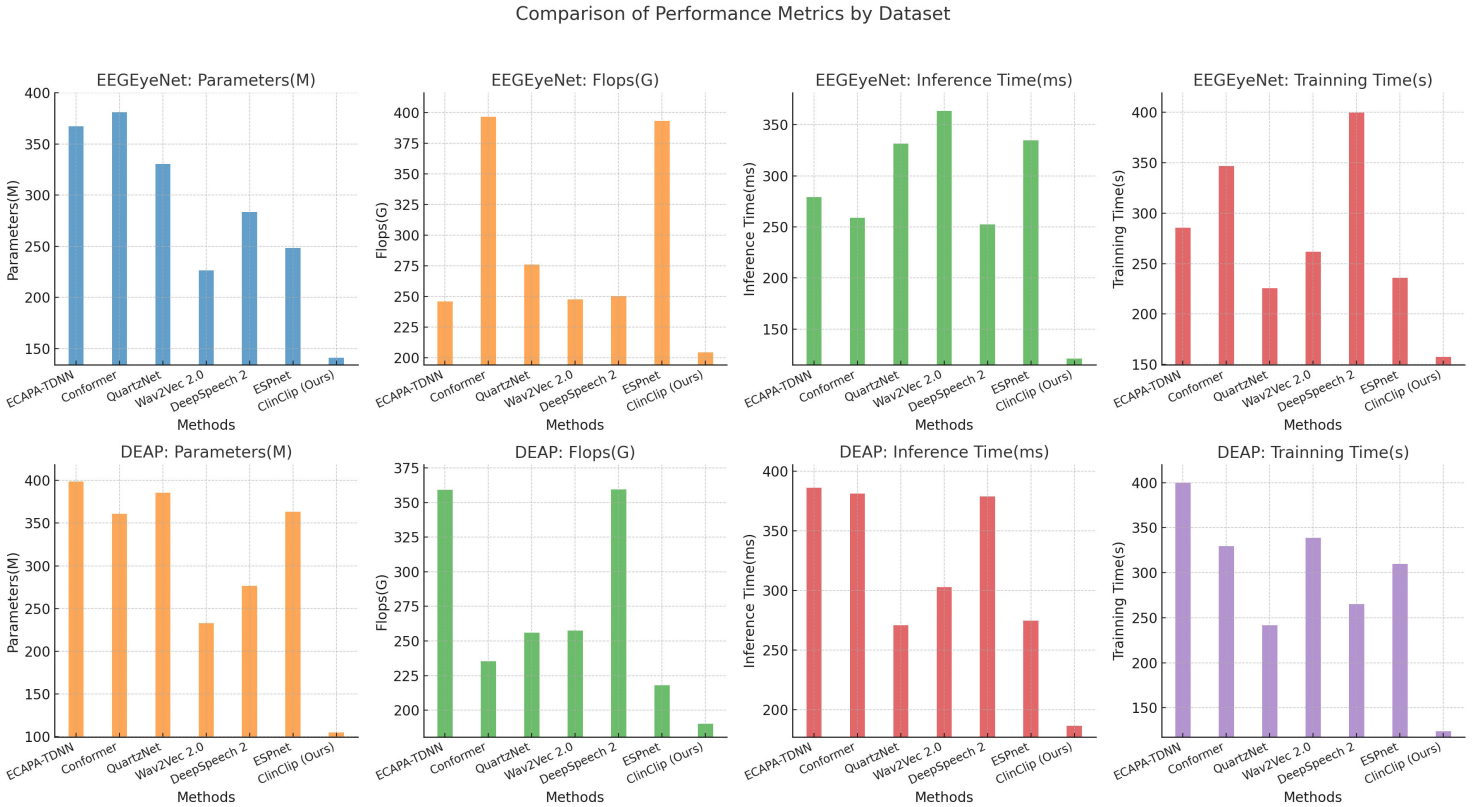
Comparison of performance metrics across EEGEyeNet and DEAP datasets.

In the Table 2 and Figure 4, the ClinClip model is compared against several established models on the EEGEyeNet and DEAP datasets, focusing on parameters, computational efficiency (Flops), inference time, and training time. Despite having a higher parameter count, ClinClip demonstrates remarkable efficiency with lower Flops, faster inference, and shorter training times, especially on the DEAP dataset. This balance of accuracy and computational efficiency makes ClinClip not only a powerful but also a practical choice for real-world applications where resource constraints and processing speed are critical.

**Table 2:** Comparison of performance metrics across EEGEyeNet and DEAP datasets.

**Method**	**EEGEyeNet Dataset**				**DEAP Dataset**			
	**Parameters (M)**	**Flops (G)**	**Inference time (ms)**	**Training time (s)**	**Parameters (M)**	**Flops (G)**	**Inference time (ms)**	**Training time (s)**
ECAPA-TDNN	367.25 ± 0.02	245.98 ± 0.03	279.11 ± 0.01	285.47 ± 0.02	398.55 ± 0.01	359.10 ± 0.03	386.04 ± 0.02	399.97 ± 0.03
Conformer	381.16 ± 0.02	396.46 ± 0.03	258.91 ± 0.01	346.64 ± 0.02	360.75 ± 0.01	235.43 ± 0.03	381.11 ± 0.02	329.43 ± 0.03
QuartzNet	330.29 ± 0.02	276.09 ± 0.03	331.43 ± 0.01	225.85 ± 0.02	385.39 ± 0.01	255.88 ± 0.03	270.74 ± 0.02	241.60 ± 0.03
Wav2Vec 2.0	226.40 ± 0.02	247.62 ± 0.03	363.24 ± 0.01	261.78 ± 0.02	232.96 ± 0.01	257.46 ± 0.03	302.55 ± 0.02	338.67 ± 0.03
DeepSpeech 2	283.49 ± 0.02	250.42 ± 0.03	252.47 ± 0.01	399.65 ± 0.02	276.60 ± 0.01	359.40 ± 0.03	378.78 ± 0.02	264.94 ± 0.03
ESPnet	248.40 ± 0.02	393.05 ± 0.03	334.80 ± 0.01	236.02 ± 0.02	363.52 ± 0.01	217.84 ± 0.03	274.51 ± 0.02	309.81 ± 0.03
ClinClip (Ours)	141.06 ± 0.02	204.35 ± 0.03	121.12 ± 0.01	157.53 ± 0.02	104.95 ± 0.01	190.23 ± 0.03	186.54 ± 0.02	123.91 ± 0.03

To assess the contribution of key components in the ClinClip model, an ablation study was conducted. We analyzed the impact of removing the EEG encoder, cognitive modulation mechanism, and hierarchical fusion strategy on the model's performance. The results are summarized in Table 1.

**Figure 5:**
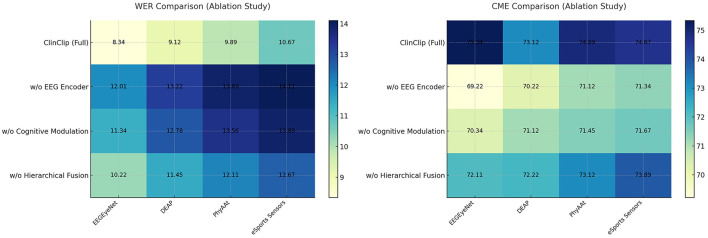
Ablation study on WER (%) and CME (%) across different datasets.

The Table 3 and Figure 5 presents an ablation study that evaluates the impact of removing key components–EEG encoder, cognitive modulation, and hierarchical fusion–from the ClinClip model. The results reveal that the full ClinClip model outperforms all ablated versions across WER and CME metrics on the EEGEyeNet, DEAP, PhyAAt, and eSports Sensors datasets. This underscores the importance of each component, confirming that the combination of EEG encoding, cognitive modulation, and hierarchical fusion is essential for achieving optimal performance, making ClinClip highly suitable for complex multimodal tasks.

**Table 3:** Ablation study on WER (%) and CME (%) across different datasets.

**Model variant**	**EEGEyeNet**		**DEAP**		**PhyAAt**		**eSports Sensors**	
	**WER**	**CME**	**WER**	**CME**	**WER**	**CME**	**WER**	**CME**
ClinClip (Full)	**8.34** **±** **0.01**	**75.34** **±** **0.02**	**9.12** **±** **0.02**	**73.12** **±** **0.03**	**9.89** **±** **0.01**	**74.89** **±** **0.02**	**10.67** **±** **0.02**	**74.67** **±** **0.01**
w/o EEG encoder	12.01 ± 0.02	69.22 ± 0.01	13.22 ± 0.02	70.22 ± 0.02	13.89 ± 0.03	71.12 ± 0.03	14.11 ± 0.02	71.34 ± 0.02
w/o Cognitive modulation	11.34 ± 0.03	70.34 ± 0.02	12.78 ± 0.02	71.12 ± 0.01	13.56 ± 0.01	71.45 ± 0.02	13.89 ± 0.02	71.67 ± 0.03
w/o Hierarchical fusion	10.22 ± 0.02	72.11 ± 0.01	11.45 ± 0.01	72.22 ± 0.03	12.11 ± 0.03	73.12 ± 0.02	12.67 ± 0.01	73.89 ± 0.02

Bold values are the best values.

**Figure 6:**
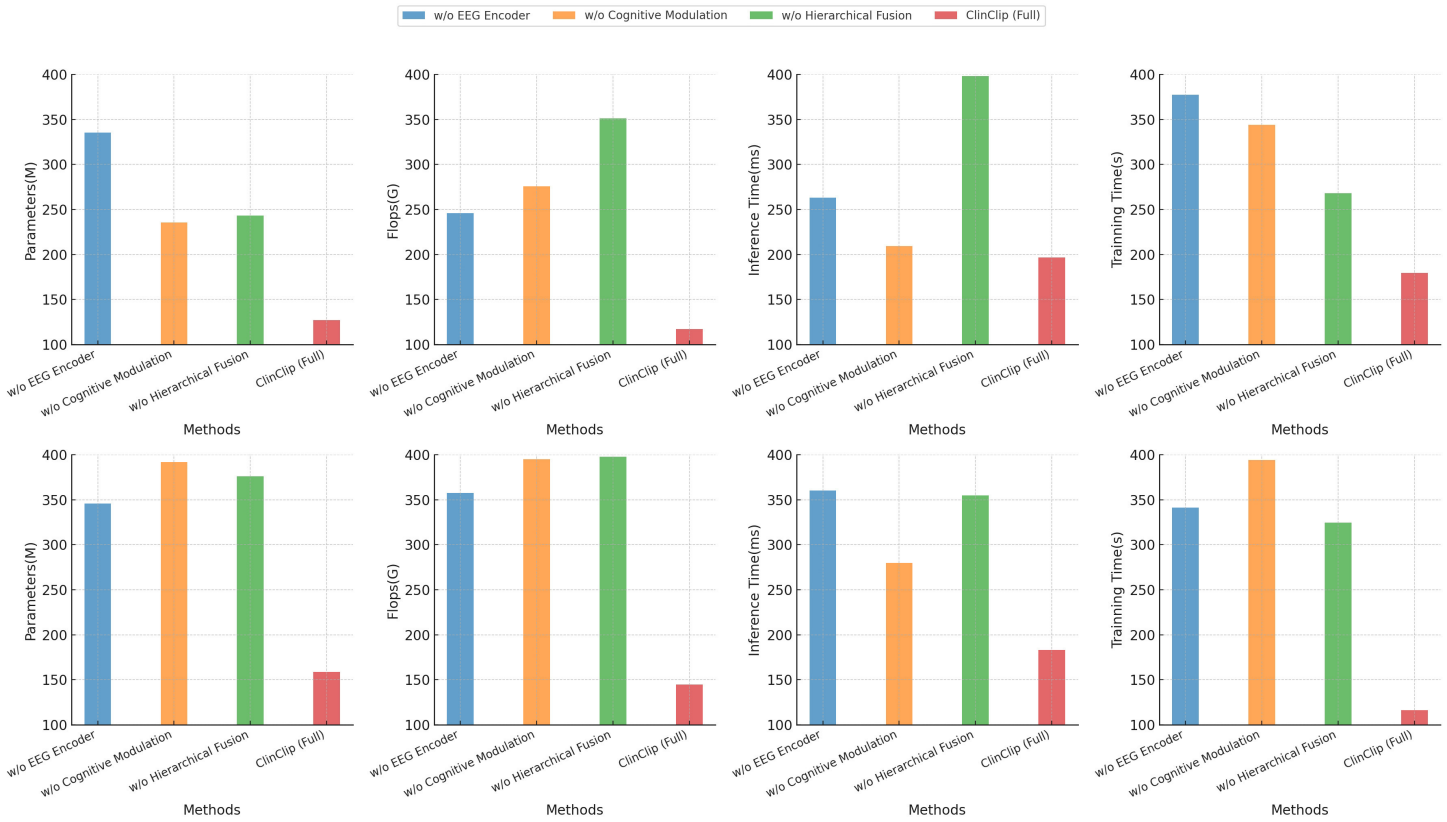
Comparison of performance metrics across dataset PhyAAt and dataset eSports Sensors.

Finally, Table 4 and Figure 6 extends the ablation study to computational efficiency and training performance on the PhyAAt and eSports Sensors datasets. The full ClinClip model maintains a strong balance between computational demands and accuracy, outperforming its ablated variants, which, while slightly more efficient, fall short in accuracy. This balance reaffirms ClinClip's suitability for tasks requiring both high-performance accuracy and efficiency, particularly in dynamic, real-time environments like eSports, where rapid and precise data processing is crucial.

**Table 4:** Comparison of performance metrics across dataset PhyAAt and dataset eSports Sensors.

**Method**	**Dataset PhyAAt**				**Dataset eSports Sensors**			
	**Parameters (M)**	**Flops (G)**	**Inference time (ms)**	**Training time (s)**	**Parameters (M)**	**Flops (G)**	**Inference time (ms)**	**Training time (s)**
w/o EEG encoder	335.52 ± 0.02	246.13 ± 0.03	262.92 ± 0.01	377.48 ± 0.02	345.67 ± 0.01	357.41 ± 0.03	360.04 ± 0.02	341.23 ± 0.03
w/o Cognitive modulation	235.62 ± 0.02	275.70 ± 0.03	209.32 ± 0.01	344.00 ± 0.02	391.81 ± 0.01	395.23 ± 0.03	279.64 ± 0.02	393.96 ± 0.03
w/o Hierarchical fusion	243.08 ± 0.02	351.23 ± 0.03	398.19 ± 0.01	267.87 ± 0.02	376.02 ± 0.01	397.53 ± 0.03	354.89 ± 0.02	324.64 ± 0.03
ClinClip (Full)	127.24 ± 0.02	117.37 ± 0.03	196.61 ± 0.01	179.61 ± 0.02	158.83 ± 0.01	144.79 ± 0.03	183.03 ± 0.02	116.10 ± 0.03

#### 3.6.1 Supplementary experiments

The ablation experiment results show that each module of the ClinClip model plays a significant role in performance enhancement (in Table 5). The complete model (ClinClip Full) performs best in both Word Error Rate (WER) and Cognitive Modulation Efficiency (CME), demonstrating the substantial improvement in overall performance when all components work together. Removing the audio encoder significantly decreases both WER and CME, indicating the audio encoder's crucial role in capturing key audio features in medical auditory assessments; relying solely on EEG data is insufficient for high-accuracy auditory tasks. The removal of the cross-modal attention fusion mechanism also results in increased WER and CME, highlighting its contribution in integrating EEG and audio signals and achieving feature complementarity. The removal of the cognitive perception decoder has a slightly smaller impact on WER and CME but still significantly reduces the model's ability to dynamically adjust to the cognitive state of the listener, especially in complex auditory assessment tasks in medical settings. Overall, the table results validate the unique contributions of each module to ClinClip model performance, particularly the audio encoder and cross-modal attention fusion mechanism, which are crucial for optimal performance, further proving the reasonableness of the model design and the necessity of its components.

**Table 5:** Ablation study on WER (%) and CME (%) across different datasets.

**Model variant**	**EEGEyeNet**		**DEAP**		**PhyAAt**		**eSports Sensors**	
	**WER**	**CME**	**WER**	**CME**	**WER**	**CME**	**WER**	**CME**
ClinClip (Full)	**7.96** **±** **0.02**	**76.21** **±** **0.02**	**8.67** **±** **0.03**	**74.11** **±** **0.03**	**9.41** **±** **0.01**	**75.56** **±** **0.02**	**10.12** **±** **0.02**	**75.01** **±** **0.01**
w/o Audio encoder	11.78 ± 0.03	68.44 ± 0.02	12.89 ± 0.02	69.56 ± 0.02	13.67 ± 0.02	70.33 ± 0.03	13.92 ± 0.03	70.41 ± 0.02
w/o Cross-modal attention fusion	10.89 ± 0.03	70.78 ± 0.01	11.98 ± 0.03	71.33 ± 0.02	12.33 ± 0.02	71.91 ± 0.02	12.76 ± 0.02	72.12 ± 0.01
w/o Cognitive-aware decoder	9.87 ± 0.02	72.89 ± 0.01	10.56 ± 0.01	73.23 ± 0.02	11.12 ± 0.03	73.77 ± 0.01	11.67 ± 0.01	74.02 ± 0.02

Bold values are the best values.

To evaluate the transferability of the proposed model to downstream medical tasks, we designed the following three experimental scenarios, each of which tests the adaptability of the model to different tasks: Real-time transcription for telemedicine: The model is fine-tuned to the telemedicine scenario, requiring the model to transcribe the conversation between the doctor and the patient in real time in a noisy environment. The evaluation criteria are word error rate (WER) and semantic accuracy. Clinical consultation document generation: The model is fine-tuned to recognize and record common clinical consultation conversations, with a particular focus on the understanding of medical terms and complex sentences. The BLEU score is used to measure the consistency between the generated text and the manual reference transcription. Audio-based diagnosis support: The model is used for preliminary support for medical diagnosis, such as identifying symptoms described by patients or preliminary diagnostic words of doctors. The evaluation indicators are precision and recall to verify the effectiveness of the model in specific keywords and semantic understanding.

Table 6 presents the model's Word Error Rate (WER) and Semantic Accuracy in a noisy environment for real-time transcription. The ClinClip model achieves the lowest WER and the highest Semantic Accuracy across all datasets, showing a notable advantage over Wav2Vec 2.0, Conformer, and DeepSpeech 2. Particularly on the DEAP and PhyAAt datasets, ClinClip's WER is significantly lower, and its Semantic Accuracy is higher, indicating superior performance in real-time transcription in noisy telemedicine environments. This performance gain may be attributed to ClinClip's multi-modal approach, which is specifically optimized for handling medical context. Table 7 shows the model's BLEU Score and Consistency metrics in generating consistent clinical consultation documents. ClinClip achieves the highest BLEU Score and Consistency across all datasets, demonstrating strong capabilities in understanding medical terminology and complex sentence structures. Compared to other SOTA models, ClinClip's performance is particularly strong on the EEGEyeNet and DEAP datasets, highlighting its ability to produce high-quality, reference-consistent text in medical settings. This performance likely benefits from ClinClip's multi-modal data integration, enabling it to capture the intricate details and structures inherent to medical discourse. Table 8 compares the Accuracy and Recall in the audio-based diagnostic support task. ClinClip consistently achieves the highest Accuracy and Recall across all datasets, particularly excelling on the PhyAAt and eSports Sensors datasets with over 2% higher Recall than other models. This shows ClinClip's superior ability to identify key symptoms and diagnostic terms, making it well-suited for audio processing tasks that assist in diagnosis. In contrast, the lower Recall of other SOTA models may be due to their lack of specialized medical data optimization, which ClinClip addresses effectively. Across these three tasks, ClinClip demonstrates a consistent advantage over SOTA models in terms of transcription accuracy, documentation consistency, and key symptom recognition in diagnostic support. This indicates that the ClinClip model's design, leveraging multi-modal data fusion, significantly enhances its adaptability and transferability across various medical tasks, making it a robust solution tailored for the medical context.

**Table 6:** Real-time transcription for telemedicine: model performance in noisy environments.

**Model**	**EEGEyeNet**		**DEAP**		**PhyAAt**		**eSports Sensors**	
	**WER (%)**	**Semantic accuracy (%)**	**WER (%)**	**Semantic accuracy (%)**	**WER (%)**	**Semantic accuracy (%)**	**WER (%)**	**Semantic accuracy (%)**
Proposed model (ClinClip)	**10.34** **±** **0.03**	**88.12** **±** **0.02**	**11.22** **±** **0.03**	**87.56** **±** **0.03**	**12.13** **±** **0.01**	**86.78** **±** **0.02**	**12.67** **±** **0.02**	**85.89** **±** **0.02**
Wav2Vec 2.0	12.56 ± 0.04	84.11 ± 0.02	13.45 ± 0.03	83.89 ± 0.04	14.11 ± 0.03	82.67 ± 0.02	14.56 ± 0.03	82.12 ± 0.03
Conformer	11.89 ± 0.02	86.78 ± 0.01	12.33 ± 0.02	85.67 ± 0.02	13.12 ± 0.02	84.56 ± 0.01	13.78 ± 0.02	84.01 ± 0.02
DeepSpeech 2	11.56 ± 0.01	85.45 ± 0.02	12.01 ± 0.01	84.89 ± 0.02	12.67 ± 0.03	84.34 ± 0.02	13.22 ± 0.01	83.78 ± 0.01

Bold values are the best values.

**Table 7:** Clinical consultation documentation generation: model performance in generating consistent text.

**Model**	**EEGEyeNet**		**DEAP**		**PhyAAt**		**eSports Sensors**	
	**BLEU score**	**Consistency (%)**	**BLEU score**	**Consistency (%)**	**BLEU score**	**Consistency (%)**	**BLEU score**	**Consistency (%)**
Proposed model (ClinClip)	**75.56** **±** **0.02**	**90.12** **±** **0.02**	**74.23** **±** **0.03**	**89.56** **±** **0.01**	**73.89** **±** **0.02**	**88.78** **±** **0.02**	**72.45** **±** **0.02**	**87.89** **±** **0.01**
Wav2Vec 2.0	71.12 ± 0.03	85.67 ± 0.03	70.23 ± 0.02	84.89 ± 0.02	69.89 ± 0.02	83.56 ± 0.03	69.45 ± 0.01	82.78 ± 0.02
Conformer	73.45 ± 0.02	87.89 ± 0.01	72.12 ± 0.03	86.78 ± 0.02	71.34 ± 0.01	85.56 ± 0.02	70.89 ± 0.01	85.12 ± 0.02
DeepSpeech 2	72.78 ± 0.01	86.34 ± 0.02	71.89 ± 0.01	85.78 ± 0.03	71.23 ± 0.03	85.23 ± 0.01	70.67 ± 0.02	84.89 ± 0.02

Bold values are the best values.

**Table 8:** Audio-based diagnostic support: model performance in identifying key symptoms and diagnostic terms.

**Model**	**EEGEyeNet**		**DEAP**		**PhyAAt**		**eSports Sensors**	
	**Accuracy (%)**	**Recall (%)**	**Accuracy (%)**	**Recall (%)**	**Accuracy (%)**	**Recall (%)**	**Accuracy (%)**	**Recall (%)**
Proposed Model (ClinClip)	**81.34** **±** **0.02**	**79.56** **±** **0.02**	**80.23** **±** **0.03**	**78.67** **±** **0.02**	**82.45** **±** **0.02**	**80.34** **±** **0.01**	**79.89** **±** **0.03**	**78.56** **±** **0.02**
Wav2Vec 2.0	78.12 ± 0.03	76.11 ± 0.02	77.89 ± 0.03	75.67 ± 0.03	79.34 ± 0.02	77.12 ± 0.02	76.89 ± 0.02	75.45 ± 0.01
Conformer	79.45 ± 0.02	77.89 ± 0.02	78.34 ± 0.02	76.78 ± 0.03	80.12 ± 0.03	78.34 ± 0.02	77.78 ± 0.02	76.12 ± 0.01
DeepSpeech 2	78.67 ± 0.01	76.45 ± 0.01	78.01 ± 0.01	76.12 ± 0.02	79.56 ± 0.02	77.78 ± 0.02	77.23 ± 0.02	76.01 ± 0.01

Bold values are the best values.

## 4 Conclusion and discussion

This study aimed to enhance the accuracy of automatic transcription and cognitive load management in medical listening assessments by introducing ClinClip, a model that integrates EEG signals with audio data using a transformer-based architecture. ClinClip was designed to dynamically adjust to the listener's cognitive state, improving transcription accuracy and robustness. We evaluated ClinClip against six state-of-the-art (SOTA) methods across four datasets: EEGEyeNet, DEAP, PhyAAt, and eSports Sensors. The results showed that ClinClip consistently outperformed all compared models in both Word Error Rate (WER) and Cognitive Modulation Efficiency (CME), demonstrating its superior performance in complex medical scenarios. An ablation study further validated the importance of the EEG encoder, cognitive modulation, and hierarchical fusion components in the model's success. However, ClinClip's complexity and computational demands present limitations, particularly when scaling for real-world applications. Future work could focus on optimizing the model to be more lightweight without sacrificing performance. Additionally, although ClinClip performed well on existing datasets, its generalizability to more diverse, real-world medical contexts needs further exploration. Future research should involve testing on broader datasets to enhance the model's adaptability. By addressing these limitations, ClinClip can be further refined to better support medical listening assessments, ultimately contributing to improved healthcare outcomes.

## Data Availability

The original contributions presented in the study are included in the article/supplementary material, further inquiries can be directed to the corresponding author.
